# The Novel Diagnostic Biomarkers for Focal Segmental Glomerulosclerosis

**DOI:** 10.1155/2014/574261

**Published:** 2014-03-26

**Authors:** Mohsen Nafar, Shiva Kalantari, Shiva Samavat, Mostafa Rezaei-Tavirani, Dorothea Rutishuser, Roman A. Zubarev

**Affiliations:** ^1^Department of Nephrology, Shahid Labbafinejad Medical Center, Shahid Beheshti University of Medical Sciences, Tehran 1666694516, Iran; ^2^Urology and Nephrology Research Center, Shahid Beheshti University of Medical Sciences, Tehran, Iran; ^3^Chronic Kidney Disease Research Center, Shahid Beheshti University of Medical Sciences, Tehran, Iran; ^4^Department of Basic Sciences, Faculty of Paramedical Sciences, Shahid Beheshti University of Medical Sciences, Tehran, Iran; ^5^Clinical Proteomics Research Center, Shahid Beheshti University of Medical Sciences, Tehran, Iran; ^6^Department of Medical Biochemistry and Biophysics, Karolinska Institute, Stockholm, Sweden; ^7^SciLifeLab, Stockholm, Sweden

## Abstract

*Background*. Focal segmental glomerulosclerosis (FSGS) is a glomerular injury with various pathogenic mechanisms. Urine proteome panel might help in noninvasive diagnosis and better understanding of pathogenesis of FSGS. *Method*. We have analyzed the urine sample of 11 biopsy-proven FSGS subjects, 8 healthy controls, and 6 patients with biopsy-proven IgA nephropathy (disease controls) by means of liquid chromatography tandem mass spectrometry (nLC-MS/MS). Multivariate analysis of quantified proteins was performed by principal component analysis (PCA) and partial least squares (PLS). *Results*. Of the total number of 389 proteins, after multivariate analysis and additional filter criterion and comparing FSGS versus IgA nephropathy and healthy subjects, 77 proteins were considered as putative biomarkers of FSGS. CD59, CD44, IBP7, Robo4, and DPEP1 were the most significant differentially expressed proteins. These proteins are involved in pathogenic pathways: complement pathway, sclerosis, cell proliferation, actin cytoskeleton remodeling, and activity of TRPC6.There was complete absence of DPEP1 in urine proteome of FSGS subjects compared with healthy and disease controls. DPEP1 acts via leukotrienes on TRPC6 and results in increased podocyte motility and proteinuria. *Conclusion*. The results suggest a panel of candidate biomarkers for noninvasive diagnosis of FSGS, while complete absence of DPEP1 might represent a novel marker of FSGS.

## 1. Introduction

Focal segmental glomerulosclerosis (FSGS) is categorized as a type of nephrotic syndrome (approximately 20% of cases of the nephrotic syndrome in children and 40% of such cases in adults) and characterized by scattered sclerosis of glomeruli in which only a segment of the capillary is affected [1–3]. The incidence of the disease is estimated as 7 per 1 million [3]. Characteristic feature of the disease is proteinuria, which implies the loss of filtration barrier in glomeruli [2, 4]. Podocyte damage that occurs by different mechanisms is considered a key factor in the pathogenesis of FSGS [5, 6]. Recent studies suggest increased levels of circulating soluble urokinase-type plasminogen activator receptor (suPAR) as one of the possible causes of podocytopathy in FSGS [7]. This protein has been announced as a novel potential diagnostic FSGS biomarker recently [8–10]. Damage to podocytes triggers apoptosis [6, 11] and foot process effacement and leads to proteinuria [12].

A wide variety of mechanisms are involved in pathogenesis of FSGS, including oxidative stress [13, 14], inflammation associated with mononuclear leukocyte recruitment [15, 16], hemodynamic abnormalities, cytoskeletal derangements, podocyte injury and apoptosis, inflammation, extracellular matrix expansion, and fibrosis [17].

FSGS is a disease entity defined by findings on traditional kidney biopsy [18, 19], which is an invasive approach, and is based on histopathological features; therefore, the search for a noninvasive biomarkers as the complementary tests in the diagnosis of glomerular diseases including FSGS seems to be necessary, particularly when renal biopsy is limited or contraindicated. Proteomics has been widely used as a platform to identify noninvasive biomarkers of health and disease status, especially in identification of potential nephropathy-associated biomarkers [20–22]. However, in spite of progresses made by proteomic experiments on tissue and urine samples in animals and patients [23, 24], novel biomarkers for improving the diagnosis of FSGS are still lacking [25]. In the present study, we used gel-free based proteomic technology in association with label-free quantification method to identify and quantify potential noninvasive biomarkers in FSGS.

## 2. Method

### 2.1. Clinical Subjects and Sample Collection

Second morning urine samples were collected from 70 patients with proteinuria on the day of biopsy, out of which 11 patients were found to have FSGS on biopsy (male = 7, female = 4, and mean age = 36.36). Patients with known secondary causes of FSGS were excluded. Eight healthy volunteers (male = 6, female = 2, and mean age = 34.5) were enrolled as healthy controls (e.g., normal renal function with neither proteinuria nor any history of chronic disease). From the non-FSGS proteinuric group, six patients with biopsy-proven IgA nephropathy (male = 5, female = 1, and mean age = 30.83) were enrolled as disease controls. The samples were collected between 2011 and 2012 at Labbafinejad Hospital and a single pathologist reported all the biopsy samples. This study was approved by the regional ethics committee in Shahid Beheshti University of Medical Sciences. Age, sex, and demographic data of the patients were noted and patients with concurrent diseases such as diabetes were excluded. Each patient was evaluated for serum creatinine, eGFR (by CKD-EPI equation), presence of hypertension, and amount of proteinuria at presentation. Sample preparation and protein extraction and digestion procedure were performed as described previously by Kalantari et al. [26].

### 2.2. Liquid Chromatography Tandem Mass Spectrometry

Liquid chromatography tandem mass spectrometry (nLC-MS/MS) analyses were performed on an Easy-nLC system coupled online to a Q-Exactive mass spectrometer (both from Thermo Scientific, Bremen, Germany). A 10 cm in-house packed silica tip column (SilicaTips New Objective Inc., Woburn, MA, USA) with Reprosil-Pur C18-AQ 3 *μ*m resin (Dr. Maisch GmbH, Ammerbuch-Entringen, Germany) was used for peptide separation. Buffers A (0.1% formic acid in water v/v) and B (0.1% formic acid in acetonitrile v/v) were applied as mobile phases. Setting of LC gradient was as follows: 5–35% buffer B in 89 min, 48–80% buffer B in 5 min, and 80% buffer B for 8 min, all at a flow rate of 300 nL/min. A temperature-controlled autosampler was used for sample injection (10 *μ*L corresponding to approximately 2.3 *μ*g of total protein). The details of MS settings were described previously [26]. All the experiments were done in duplicate (50 runs in total).

### 2.3. Protein Identification and Quantification

Mascot 2.3.0 search engine (Matrix Science Ltd., London, UK) was used for searching the extracted tandem mass spectra against concatenated SwissProt protein database (human taxonomy). Carbamidomethylation of cysteines was considered as a fixed modification and deamidation of asparagine and glutamine and oxidation of methionine were set as variable modifications. Mass tolerance was set to 10 ppm for the precursor ion and 0.05 Da for the fragment ions and up to two missed tryptic cleavages were allowed. Only proteins identified with at least two unique peptides with a significant score and at 0.25% false discovery rate (FDR) were considered for further quantification.

Label-free peptide and protein quantification was performed using Quanti software (an in-house developed software package) [27]. The sum of the abundances of all unique peptides (the areas of the chromatographic peaks) of a protein was used as the protein abundance value.

### 2.4. Statistical Analysis

Multivariate statistical analyses were performed using SIMCA (SIMCA-p 13.0, Umetrics, Umeå, Sweden). Unsupervised principal component analysis (PCA) [28] was performed without consideration of group information for observing the overview of the data structure, detecting the clusters of the data, and identifying the outliers if any.

Partial least squares (PLS) [29] analysis was applied in order to reach a predictive model to discriminate FSGS patients and healthy controls based on proteomics data obtained by PCA.

For classification and identification of proteins separating FSGS from disease control (IgAN) patients, we used orthogonal projection to latent structures discriminant analysis (OPLS-DA) [30]. To avoid overestimation, sevenfold cross-validated scores were calculated for PLS and OPLS-DA models [31].

### 2.5. Protein GO-Term Enrichment and Pathway Analysis

In order to characterize properties of the proteins in the dataset and also to detect the enriched cellular component, molecular function, and biological process, gene ontology enrichment was performed using DAVID open-source software tool [32]. A cutoff for enrichment score for DAVID software result was set at 1.3 and the redundant hits were excluded.

## 3. Results

### 3.1. Clinical and Pathological Characteristics of Patients

Clinical and laboratory data of patients are presented in Table 1. Eleven patients with biopsy-proven FSGS, six patients with IgA nephropathy (IgAN), and 8 healthy volunteers were enrolled. 24 h urine collection was used to estimate the amount of protein excretion. The mean 24 h protein excretion was 3010 and 3980 mg/day and the mean eGFR (by CKD-EPI equation) was 62.15 and 46.87 cc/min/1.73 m^2^ among FSGS and IgAN patients, respectively.

### 3.2. Unsupervised Statistical Analysis by PCA

**Figure 1:**
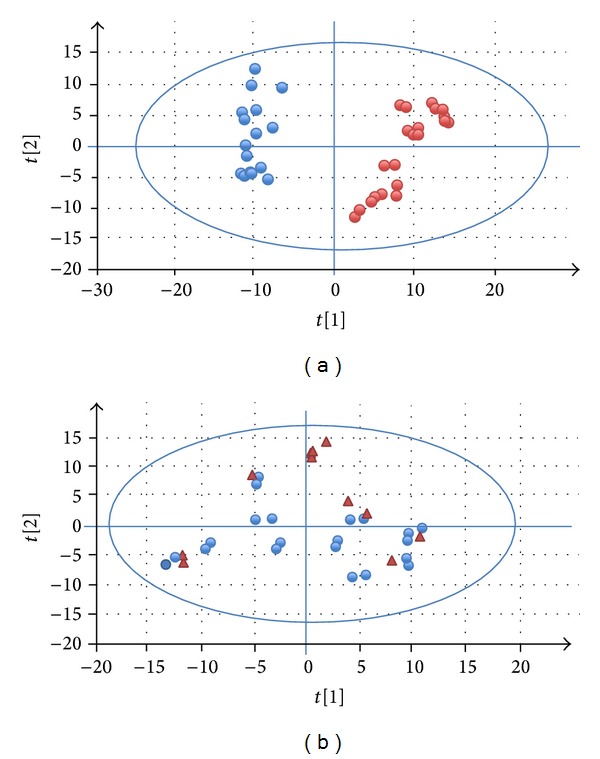
Score plot of PCA. (a) Blue circles represent healthy controls and red dots represent FSGS patient samples. (b) Blue dots represent FSGS patient samples and red triangles represent IgAN patient samples. Each of the samples was analyzed with two technical replicates.

A total number of 389 unique proteins were identified and quantified by nLC-MS/MS (Table S1; see Supplementary Material available online at http://dx.doi.org/10.1155/2014/574261). Two clusters were obtained by PCA for FSGS patients/healthy controls (Figure 1(a)) but not for FSGS/IgAN patients (Figure 1(b)). The clustering illustrated in Figure 1(a) implies large difference between patients and healthy controls detectable by PC1 and PC2. Lack of clustering in Figure 1(b) implies relatively small difference in urine proteome among FSGS and IgAN subjects. There were no outliers on both PCA score plots, which means that no confounding factor affected our study. Out of the 389 proteins used for clustering, 154 proteins showed statistically significant abundance changes, and thus they were identified to be the most important markers responsible for the observed clustering in Figure 1(a) (Table S2).

### 3.3. Supervised Statistical Analysis

**Figure 2:**
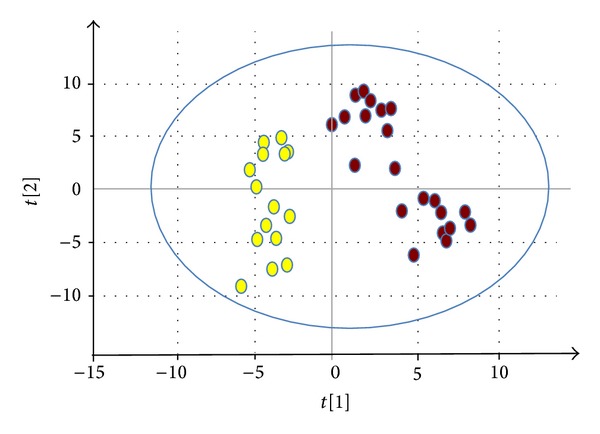
Predictive model (FSGS/normal controls). Partial least squares discriminant analysis (PLS-DA) model for discrimination of healthy controls (yellow circles) and FSGS (red dots) patient samples.

Based on the significant proteins obtained from PCA results of FSGS patients and healthy control proteomes, a predictive model could be built to distinguish between these two clinical conditions (Figure 2). This model was constructed using PLS (partial least squares) method [29]. The predictive (*Q* ^2^) and fitness values (*R* ^2^) were 0.393 and 0.366, respectively with 98% accuracy. By the PLS model, 132 proteins significantly contributed to the FSGS patient/healthy control discrimination. These proteins are listed in Table S3. After applying an additional filter criterion where only proteins with a fold change of 1.5 or higher were retained, 90 proteins remained, of which 20 proteins were upregulated (overrepresented) in disease state and 70 proteins were downregulated or underrepresented (Table S4). This panel of proteins was used for further gene set enrichment and pathway analysis by DAVID.

**Figure 3:**
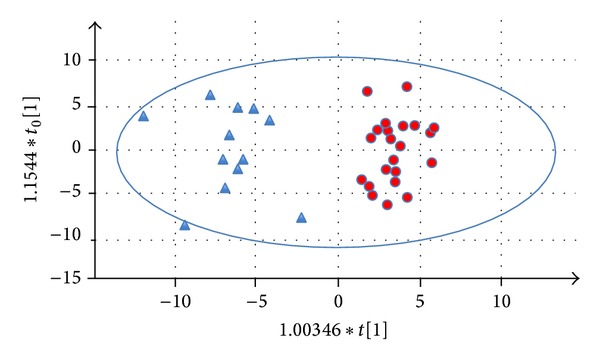
Predictive model (FSGS/IgAN). Orthogonal projections to latent structures discriminant analysis (OPLS-DA) model for discrimination of FSGS (red dots) and IgAN (blue triangles) patient samples.

OPLS-DA model gave *Q* ^2^ = 0.825 and *R* ^2^ = 0.374 for the discrimination of FSGS and IgAN patients (Figure 3) and showed a predictive accuracy of 100%. The significant proteins responsible for discrimination of FSGS and IgAN are listed in Table S5. After applying an additional filter criterion where only proteins with a fold change of 1.5 or higher were retained, of 35 remaining proteins, 11 proteins were upregulated (overrepresented) in FSGS compared to IgAN patients and 25 proteins were downregulated or underrepresented (Table S6).

Seventy-eight proteins were uniquely different between FSGS patients and healthy controls and were not in discriminative protein list when comparing FSGS/IgAN. They could be considered as specific biomarkers for FSGS (Table S7). The top twelve proteins (six most positively and six most negatively presented ones) are presented in Table 2 as putative diagnostic biomarkers of FSGS.

We also compared whole protein profile of FSGS patients and healthy subjects in order to find qualitative biomarker candidates. DPEP1 (dipeptidase 1) was the only protein isolated in all healthy subjects while it was not detected in FSGS patient samples at all. Since DPEP1 was also detected in IgA patients, it is suggested as a potential specific biomarker for FSGS.

Gene set enrichment analysis yielded eight significant biological processes (Table S8). The most significant process was “response to wounding” (*p* = 5.2 × 10^−9^). The significant cellular components and molecular functions are listed in Tables S9 and S10. Pathway analysis using DAVID with the KEGG database showed two major pathways: “complement and coagulation cascades” (*p* = 2 × 10^−7^) and “lysosome” (*p* = 8 × 10^−4^) (Table S11).

## 4. Discussion

Focal segmental glomerulosclerosis (FSGS) is a glomerular nephropathy and also one of the causes of nephrotic syndrome in children and adults [33]. Environmental toxins, genetic factors, infectious agents, haemodynamic abnormalities, or other types of nephritis are some of the known risk factors for FSGS [1]. So far, diagnosis of FSGS relies on renal biopsy, which is an invasive traditional diagnostic approach [4, 18]. Seeking for potential diagnostic protein biomarkers available in urine via high-resolution proteomic tools can provide a noninvasive way for diagnosis of FSGS. By nLC-MS/MS analysis, we define a panel of potential biomarkers in FSGS patients that could be used not only as a diagnostic model but also as an extension of our knowledge about the pathogenesis of FSGS. Comparing urinary proteome panel of FSGS patients with that of IgA nephropathy highlighted the disease specific biomarkers.

Ninety differently expressed proteins remained significant between patients and healthy controls after multivariate statistical analysis, PLS, and additional filtration based on fold change, of which seventy-eight proteins were unique and specific for FSGS.

The most drastic change (24.4-fold decrease in expression) in this group belonged to CD59, which is a glycophosphoinositol- (GPI-) anchored inhibitor of the membrane attack complex (MAC) in complement pathways. This protein in a lipid-tailed status is expressed on blood cells and endothelial and epithelial cells; however, soluble lipid-free forms of CD59 have also been reported in human body fluids [34]. While the main purpose of MAC is to attack invading microorganisms and cell lysis, CD59 inhibits its formation to protect host cells against self-destruction [34–36]. Turnberg et al. in 2006 revealed the direct relationship between CD59 and adriamycin nephropathy, a model of FSGS, in CD59-deficient mice [37]. They stated that lack of CD59 led to greater glomerular and tubulointerstitial injury, as CD59 protects glomerulus against MAC. In addition, Arora et al. reported the decreased expression of CD59 on erythrocytes and biopsy specimens of FSGS patients [38]. Substantial underrepresentation of urinary CD59 in FSGS patients compared with normal individuals in our dataset is consistent with the former studies and verifies the implication of complement system in pathogenesis of FSGS.

CD44, which holds the second rank of changes (23-fold) in the list of top candidates obtained from PLS model, is a type I transmembrane glycoprotein that plays a role in cell-matrix interaction and cell adhesion and migration [39]. Data have suggested that activated parietal epithelial cell demonstrated increased expression of CD44 in biopsy samples, which was correlated with sclerosis [40]. The main ligand of CD44 is hyaluronic acid (HA), but it also interacts with collagen, laminin, fibronectin, and osteopontin as ligands [20]. Fatima et al. [41] recently suggested the elevated expression of CD44 in human renal biopsy as a marker for activated parietal epithelial cells in patients with recurrent FSGS, and Nakamura et al. [42] also reported a positive correlation between upregulation of CD44, hyaluronic acid, and osteopontin in biopsy specimens with early stage of the crescent formation in human crescentic glomerulonephritis. In contrast to the abovementioned studies, we reported decreased urinary excretion of CD44 in FSGS patients compared with healthy controls, which could be due to different specimens used for analysis (kidney tissue versus urine).

IBP7 (insulin-like growth factor-binding protein 7), coded by a gene named IGFBP7, is a protein secreted by podocytes with a possible regulatory function on cell cycle [43]. Since this protein regulates cell growth in cancer cells [44, 45], it is possible that IBP7 may be associated with cell cycle regulation. Previous finding suggests that IBP7 may contribute to the podocyte response to injury. Matsumoto et al. demonstrated increased IGFBP7 expression in cultured injured podocytes after exposure to TGF-*β* and also in mouse with HIV associated nephropathy [43]. Moreover, Brunisholz et al. have proposed IBP7 as a prognostic urinary biomarker for acute kidney injury (AKI) [46], which adds the importance of this protein for future analysis. Drastic underrepresentation (13-fold change) of this protein in our dataset might be due to impairment of cell cycle, proliferation and differentiation signaling in sclerotic glomeruli, diminished free filtration of IGFBP7 (33 kDa) across filtration barrier due to decreased filtration of small molecules in FSGS [47], or differences between in vitro and in vivo expressions of this protein.

In addition to abovementioned peptide, the expression of uromodulin, granulin, and proactivator peptide has been downregulated in FSGS subjects compared with healthy controls. Diminished expression of uromodulin was reported in different types of renal diseases such as diabetic nephropathy and chronic kidney disease (CKD) and may indicate pathophysiological changes in CKD [48]. Granulin is a growth factor, which plays a role in cell growth regulation, innate immunity, and wound healing. Its upregulated expression in plasma was reported to be related to the activity of lupus nephritis via macrophage activation [49]. However, the significance of granulin downregulation (11-fold change) in urine sample of FSGS patients is not obvious. There was a 9.73-fold decrease in expression of proactivator polypeptide (SAP), which is involved in lysosomal pathway and sphingolipid degradation, and its downregulation might lead to lysosomal accumulation of sphingolipids and inflammation in surrounding tissue as it will be discussed later [50].

TRFE (transferrin) is a negative acute-phase protein and is the most important glycoprotein for transport of iron in human body [51]. Increased urinary transferrin excretion has been observed in nephrotic syndrome [52]. Overrepresentation of this protein in our dataset was in accordance with Li et al. and Shui et al. findings [25, 53] on the correlation of urinary transferrin excretion and glomerulosclerosis.

Due to disease-specific changes of glomerular permeability [47], different patterns of plasma proteins including transferrin, *α*_1_ antitrypsin (A1AT), and antithrombin III would be lost in urine of patients with nephrotic syndrome and might not be specific for FSGS as they could be detected in nephrotic range proteinuria secondary to any etiology [54].

Our data demonstrated a 5.64-fold change in urine ApoA-I in FSGS subjects. The synthesis of ApoA-I is increased with the severity of nephrotic syndrome [55]. Lipid-free ApoA-I is excreted in urine [56]. Recently, a modified form of ApoA-I in urine was shown to be correlated with recurrent FSGS after transplantation and was identified by Lopez-Hellin et al. as a potential biomarker of FSGS relapse [57].

A1AG1 (alpha-1-acid glycoprotein 1), also known as orosomucoid-1, is a heavily glycosylated serum protein that has the capacity to bind and transport basic and neutral molecules [58]. This protein is known as an acute-phase protein that has immunomodulatory as well as anti-inflammatory effect and is also a marker for inflammatory diseases and endothelial injury [59–61]. Increased urinary alpha-1-acid glycoprotein has been reported to be associated with kidney injury caused by radiation therapy, trauma, and type II diabetes [62]. Urine A1AG1 expression is increased among our FSGS patients, which might indicate an active inflammatory process and glomerular injury [63].

Roundabout homolog 4 (Robo4), a member of robo receptor family expressed on epithelial cells, binds to slits and is involved in organogenesis including that of the kidney [64]. Interaction of Robo/slit can change cell motility directly and lead to reorganization of the actin cytoskeleton [65]. Robo4 through its downstream signaling molecules, Cdc 42 and Rac 1, is involved in actin cytoskeleton remodeling and filopodia [66]. Increased expression of Robo4 and therefore podocyte motility may result in proteinuria [67]. Since foot process effacement in sclerosis process is related to derangements in podocyte actin cytoskeleton [68], overrepresentation of Robo4 in FSGS patients as compared to healthy controls (a 3-fold change) in the present study may suggest its involvement in glomerulosclerosis by affecting the actin cytoskeleton.

DPEP1 (dipeptidase 1) is a kind of kidney membrane-bound enzyme that is mostly expressed in the proximal convoluted tubules [69]. It hydrolyzes a wide range of dipeptides and is also implicated in the renal metabolism of glutathione and its conjugates including leukotrienes [70]. Cysteinyl leukotrienes such as LTC4, LTD4, and LTE4 are well-known mediators for inflammatory response and are involved in acute and chronic inflammatory conditions such as asthma and glomerulonephritis, and DPEP has an important role in elimination of leukotrienes by conversion of LTD4 to LTE4 [71]. LTC4 and LTD4 have G-protein coupled receptors on podocytes. Activation of these receptors via PLC-*β* pathway results in activation of TRPC6. Increased activity of TRPC6 and thus intracellular calcium causes changes in actin cytoskeleton and FSGS [72]. Complete urinary absence of this protein may lead to FSGS by overactivity of TRPC6. To our knowledge, nonexistence of DPEP1 in the urine of FSGS patients, which was present in healthy and disease controls' urine sample, is reported for the first time in the present study and could serve as a novel marker associated with FSGS. Future metabolomics studies on the concentration level of LTC4/LTD4 and TRPC6 activity can evaluate the hypothesis of implication of DPEP1 deficiency in the pathogenesis of FSGS and a role for antileukotrienes (e.g., indomethacin) in treatment of FSGS.

Enrichment of A1AT in the six out of eight enriched biological processes by DAVID software in gene set enrichment analysis hints on its important role in pathogenesis of FSGS that can be further investigated.

Pathway analysis illustrated two major pathways: “complement and coagulation cascades” and “lysosome.” The correlation between complement cascade elements including C3 and glomerulosclerosis has been reported previously [37], and our findings also support the association of this pathway and glomerulosclerosis.

With progression of FSGS, the increasing amount of filtered proteins must be reabsorbed in proximal tubule via megalin-mediated endocytosis. The endocytosed proteins would undergo lysosomal degradation. Defects in lysosomal protein degradation result in protein accumulation and inflammation and fibrosis in the surrounding tissues [50].

Teschner et al. proposed decreased activity of intraglomerular proteinases including lysosomal proteinases as an important initiating hallmark of glomerulosclerosis [73]. Six lysosomal enzymes enriched lysosomal pathway in our analyses and all of them were underrepresented in the disease state. Loss of lysosomal enzymes in FSGS might suggest the insufficient capacity of proteinases (whether glomerular or tubular) to degrade proteins and might have a role in pathogenesis of FSGS.

This study provides novel data on urine proteome in FSGS and the involved pathways that might have a role in the pathogenesis of the disorder. However, due to small number of cases in this cohort, larger and more detailed cohort is required to confirm the significance of our findings.

## 5. Conclusion

We demonstrated the differences between urine proteome among healthy subjects and FSGS patients, which may be used as a diagnostic tool. Interestingly, we found the complete absence of DPEP1 in proteome panel of FSGS subjects by nLC-MS/MS that suggests it as a novel noninvasive diagnostic biomarker candidate. DPEP1 by its indirect activating role on TRPC6 causes derangement in actin cytoskeleton of podocytes and thus proteinuria. In addition, the increased activity of complement pathway and the defects in lysosomal pathway represent the pathophysiologic events corresponding to kidney inflammation and injury. We represent a potential noninvasive diagnostic marker with possible pathogenic role among FSGS patients; further studies should address the utility of this marker as an early diagnostic or therapeutic target.

## Supplementary Material

A total number of 389 unique proteins were identified and quantified by nLCMS/MS (Table S1). Out of the 389 proteins used for clustering, 154 proteins showed statistically significant abundance changes and are presented in Table S2. By the PLS model, 132 proteins significantly contributed to the FSGS patient/healthy control discrimination. These proteins are listed in Table S3. After applying an additional filter criterion, where only proteins with a fold change of 1.5 or higher were retained, 90 proteins remained, of which 20 proteins were upregulated (overrepresented) in disease state and 70 proteins were downregulated, or under-represented as shown in Table S4. The significant proteins discriminating FSGS and IgAN are listed in Table S5. After applying an additional filter criterion, where only proteins with a fold change of 1.5 or higher were retained, of 35 remaining proteins, 11 proteins were upregulated (overrepresented) in FSGS compared to IgAN patients and 25 proteins were downregulated, or under-represented (Table S6). Seventy-eight proteins were uniquely different between FSGS patients and healthy controls that are presented in Table S7. Gene set enrichment analysis yielded eight significant biological processes as shown in Table S8. The significant cellular components and molecular functions are listed in Tables S9 and S10. Pathway analysis using DAVID with the KEGG database showed two major pathways, “Complement and coagulation cascades” (*p*=2×10^7^) and “lysosome” (*p*=8×10^4^) (Table S11).

## Figures and Tables

**Table 1:** Demographic and laboratory characteristics of patients with focal segmental glomerulosclerosis and IgA nephropathy.

Patient's code	Age (yr)	Sex	eGFR (cc/min/1.73 m^2^)	Proteinuria (mg/day)	TA/IF	Disease
1	29	M	34.61	2031	<10%	FSGS
2	46	M	34.64	5000	30%	FSGS
3	19	M	145.76	4500	30%	FSGS
4	61	M	46.52	2590	<10%	FSGS
5	37	F	78.51	1400	<10%	FSGS
6	36	F	60.52	2710	20%	FSGS
7	37	F	42.01	710	30%	FSGS
8	30	M	38.76	2925	40%	FSGS
9	58	F	70.48	4373	<10%	FSGS
10	18	M	85.17	11000	<10%	FSGS
11	29	M	73.73	7000	10%	FSGS
12	29	M	8.58	6000	80%	IgAN
13	42	M	79.52	6420	10%	IgAN
14	29	M	15.57	7020	80%	IgAN
15	28	M	16.11	2330	80%	IgAN
16	23	F	63.65	800	20%	IgAN
17	34	M	97.76	1310	10%	IgAN

**Table 2:** Putative specific biomarkers for FSGS.

Protein ID	Protein name	Biological process	Cellular compartment	Molecular function	Fold change (FSGS/normal)	Up/downregulation
CD59	CD59 glycoprotein	Blood coagulation/cell surface receptor signaling pathway/negative regulation of apoptotic process	Anchored to external side of plasma membrane	Potent inhibitor of the complement membrane attack complex (MAC) action	24.41	↓

CD44	CD44 antigen	Wound healing involved in inflammatory response/negative regulation of apoptotic process	External side of plasma membrane	Blood group antigen receptor	22.95	↓

IBP7	Insulin-like growth factor-binding protein 7	Negative regulation of cell proliferation/regulation of cell growth	Extracellular space	Insulin-like growth factor-binding	13.06	↓

UROM	Uromodulin	Negative regulation of cell proliferation/regulation of ion homeostasis	Apical plasma membrane/extracellular space	Calcium ion binding	12.63	↓

GRN	Granulin	Signal transduction/positive regulation of epithelial cell proliferation	Extracellular space	Growth factor activity	11.08	↓

SAP	Proactivator polypeptide	Regulation of lipid metabolic process/regulation of MAPK cascade	Extracellular space/lysosomal membrane	Enzyme activator activity/lipid binding	9.73	↓

TRFE	Serotransferrin	Transferrin transport/cellular iron ion homeostasis	Apical plasma membrane/basal plasma membrane/extracellular region	Ferric iron binding	15.15	↑

A1AT	Alpha-1-antitrypsin	Regulation of proteolysis/response to cytokine stimulus/blood coagulation	Extracellular space	Serine-type endopeptidase inhibitor activity	11.52	↑

ApoA-1	Apolipoprotein A-I	Negative regulation of cytokine secretion involved in immune response/negative regulation of inflammatory response	Spherical high-density lipoprotein particle/secretory granule	High-density lipoprotein particle binding	5.64	↑

ANT3	Antithrombin-III	Blood coagulation/negative regulation of inflammatory response/response to nutrient/regulation of proteolysis	Extracellular space/plasma membrane	Serine-type endopeptidase inhibitor activity	5.18	↑

A1AG1	Alpha-1-acid glycoprotein 1	Regulation of immune system process/acute-phase response	Extracellular space	Functioning as transport protein in the blood stream	3.85	↑

Robo4	Roundabout homolog 4	Cell differentiation/negative regulation of cell migration	External side of plasma membrane	Receptor activity	3.09	↑

DPEP1*	Dipeptidase 1	Leukotriene metabolic process/negative regulation of apoptotic process	Extracellular space/anchored to membrane	Metallodipeptidase activity	—	—

*DPEP1: this protein is a qualitative biomarker. All healthy subjects had this protein while it was not detected in FSGS samples at all.
